# Gut microbiota and pathobiont exposure influences disease incidence in non-obese diabetic mice

**DOI:** 10.3389/fmicb.2026.1844128

**Published:** 2026-07-23

**Authors:** Amber L. Russell, Benjamin Olthoff, Chunye Zhang, Cat Lutz, Craig L. Franklin, Aaron C. Ericsson

**Affiliations:** 1 Department of Pathobiology and Integrative Biomedical Sciences, College of Veterinary Medicine, University of Missouri, Columbia, MO, United States; 2 Comparative Medicine Program, University of Missouri, Columbia, MO, United States; 3 The Jackson Laboratory, Bar Harbor, ME, United States; 4 University of Missouri Mutant Mouse Resource and Research Center (MU MMRRC), University of Missouri, Columbia, MO, United States; 5 University of Missouri Metagenomics Center (MUMC), University of Missouri, Columbia, MO, United States

**Keywords:** diabetes, Helicobacter, MHV, microbiome, NOD mice, segmented filamentous bacteria (SFB)

## Abstract

While the non-obese diabetic (NOD) mouse is the most widely used animal model of type 1 diabetes (T1D), it suffers from poor reproducibility in disease incidence often attributed to variables in the environment, including the gut microbiota (GM). Prior research suggests a protective effect of segmented filamentous bacteria (SFB) on disease incidence, but it is unclear whether other pathobiont organisms or resident GM affect disease incidence. The objectives of the current study were to determine the effect of supplier-origin GMs and three different microbial challenges (SFB, *Helicobacter hepaticus*, and Mouse Hepatitis Virus [MHV]) on early-stage insulitis and lifelong disease incidence in NOD mice. The fecal microbiome was assessed pre- and post-disease onset to identify shifts in composition and predicted function of the GM. Results show that all three microbes influence T1D incidence and insulitis severity. Overall, SFB, MHV, and a high-richness microbiome were associated with lower disease incidence, while *H. hepaticus* and a low-richness microbiome were associated with higher disease incidence. *H. hepaticus*, but not SFB or MHV, was associated with significant changes in beta-diversity of the GM. While immune outcomes were not included, these findings provide guidance on microbes affecting disease incidence in NOD mice and evidence that such microbes may contribute to poor reproducibility in NOD mice or other mouse models.

## Highlights

- Segmented filamentous bacteria, mouse hepatitis virus, and high-richness microbiome are associated with higher disease incidence in NOD mice.
- *Helicobacter hepaticus* and low-richness microbiome are associated with increased disease incidence.
- *Helicobacter hepaticus*, but not SFB or MHV, are associated with significant effects on the microbiome.
- These findings will allow researchers to refine, troubleshoot, and optimize studies using NOD mice, and suggest cognate taxa found in humans may also affect disease risk.

## Introduction

Type 1 diabetes (T1D) is a chronic autoimmune disease affecting roughly 9.5 million people worldwide (Ogle et al., 2025) and predicted to increase in prevalence (Gregory et al., 2022). Disease is characterized by immune-mediated destruction of insulin-producing beta cells in the pancreatic islets. While the precise etiology of T1D is unclear, disease incidence in humans is influenced by both genetic (Desai et al., 2006; Pociot and Lernmark, 2016) and environmental (Rewers and Ludvigsson, 2016) factors. Among the latter, evidence suggests that prior bacterial or viral infection (Filippi and von Herrath, 2008), as well as the resident gut microbiome (GM) (de Goffau et al., 2014; Vatanen et al., 2018), may influence susceptibility to T1D. However, a direct cause-and-effect relationship of specific microbiological factors and T1D remains elusive due to the genetic and environmental variability present within the human population (Chatzigeorgiou et al., 2009). In order to eliminate or reduce these potentially confounding variables and investigate disease mechanisms with potential translational value, animal models are often used.

The non-obese diabetic (NOD) mouse is the most frequently used animal model of T1D due to its polygenetic inheritance of spontaneous insulin-dependent diabetes mellitus (IDDM), similar to that seen in the human disease (Chatzigeorgiou et al., 2009; Chen Y. G. et al., 2018). While the NOD mouse phenotype is robust overall, inter-institutional variability in incidence and time of onset has been reported (Shoda et al., 2005; Baxter et al., 1991). Reported effects of diet (Funda et al., 1999; Mariño et al., 2017), water treatment (Sofi et al., 2014; Wolf et al., 2014), and bedding (Fahey et al., 2017) on both the GM and disease incidence in NOD mice suggest these and other institution-dependent effects on the GM may influence the model phenotype. Studies using germ-free and antibiotic-treated mice show that full or partial ablation of the GM exacerbates insulitis (Alam et al., 2011) and accelerates development of autoimmune diabetes in NOD mice (Brown et al., 2016; Candon et al., 2015; Fernandez Trigo et al., 2024), suggesting an overall protective effect of the native GM. Similarly, fecal microbiota transfer (FMT) from NOD donor mice to non-obese resistant (NOR) recipients induces insulitis (Brown et al., 2016), while FMT from disease-resistant MyD88-deficient NOD donor mice to NOD recipients is protective (Peng et al., 2014), suggesting gut dysbiosis contributes to disease progression. Prospective studies of bacteria associated with reduced disease incidence show that empirically selected bacteria can induce compositional changes in the GM and delay disease onset (Hanninen et al., 2018; Marietta et al., 2022).

Additionally, specific disease-modifying organisms (DMOs) commonly found in rodent colonies (Albers et al., 2023) have been associated with differential disease incidence in NOD mice, including segmented filamentous bacteria (SFB, *Candidatus Savagella*) (Fahey et al., 2017; Kriegel et al., 2011; Rouland et al., 2022), Mouse Hepatitis Virus (MHV) (Wilberz et al., 1991), and *Helicobacter hepaticus* (De Riva et al., 2017). SFB, a potent inducer of Th17 responses (Goto et al., 2014; Ivanov et al., 2009; Lécuyer et al., 2014), and *H. hepaticus*, a similarly potent inducer of Th1 responses (Morrison et al., 2013; Myles et al., 2007; Whary et al., 1998), are associated with reduced (Fahey et al., 2017; Kriegel et al., 2011; Rouland et al., 2022) and increased (De Riva et al., 2017) disease in NOD mice, respectively. MHV is associated with protection from T1D in NOD mice (Wilberz et al., 1991), however, as with SFB and *H. hepaticus*, these are purely associations and none of these studies prospectively, or selectively, tested the effect of each microbe on disease incidence. Moreover, the additive effects of co-infection with DMOs believed to mitigate or exacerbate disease are not clear. Lastly, there is also a significant sex bias in NOD mice, with female mice showing higher incidence and more severe insulitis (Bao et al., 2002). Studies using both sexes have reported that experimental treatment can intensify or reduce the sex-dependent difference in T1D incidence (Candon et al., 2015), potentially through a GM-dependent mechanism (Yurkovetskiy et al., 2013).

The current study was designed to determine the individual or combined influence of SFB, *H. hepaticus*, and MHV, in the context of different low- or high-richness specific pathogen-free (SPF) GMs, on early-stage insulitis and overall disease incidence of male and female NOD mice. Toward this end, three separate colonies of genetically identical NOD mice harboring distinct GMs were created via embryo transfer rederivation and used to generate experimental mice inoculated at weaning with one or more DMOs in a fully crossed study design. Separate cohorts of mice containing equivalent numbers of males and females were aged to 16 weeks (*n* = 12 mice/sex/GM/DMO) to assess early stages of disease via histopathology, or up to 52 weeks (*n* = 8 mice/sex/GM/DMO) to assess disease incidence. Blood glucose levels (BGL) were monitored twice weekly beginning at 10 weeks of age and mice were euthanized if BGL was greater than 300 mg/dL for two consecutive tests. Additionally, fecal samples were collected at weaning and at 16 weeks for 16S rRNA amplicon sequencing to determine the effect of pathobiont exposure on the composition of the GM prior to disease development, and to infer functional differences between groups based on differences in taxonomic composition.

## Research design and methods

### Colony establishment

Male and female NOD/ShiLtJ mice were generously provided by The Jackson Laboratory (Bar Harbor, ME). Complex microbiota targeted rederivation was performed at the University of Missouri Mutant Mouse Resource and Research Center (MU MMRRC) using a previously described method (Hart et al., 2018). Briefly, NOD/ShiLtJ embryos were transferred into pseudopregnant CD-1 surrogate dams harboring either low-richness gut microbiome (GM^Low^) originating from Jackson Laboratory mice, or high-richness microbiome (GM^High^) originating from Inotiv (previously Envigo, Indianapolis, IN, USA) mice, to generate NOD/ShiLtJ (NOD hereafter) offspring harboring those specific microbiomes. Colonies of CD-1 mice harboring the two different supplier-origin microbiomes are maintained as a resource of the MU MMRRC, and have been described in detail (Ericsson et al., 2025). The resulting NOD offspring were subsequently bred to generate the NOD colonies used herein. A subset of GM^Low^-colonized NOD mice were inoculated via gastric gavage with 100 μL of SFB inoculum at 3–4 weeks of age. SFB inoculum was isolated and purified from naturally colonized BALB/cAnNHsd mice (Inotiv) using a previously published method (Ericsson et al., 2015). Those SFB-colonized GM^Low^ NOD mice were then bred to produce the GM^Low^+SFB colony. GM^High^-colonized mice were not supplemented with SFB as SFB are already present in that microbiome. Feces of mice from all colonies were screened to confirm presence or absence of SFB, and all experimental mice were confirmed SFB-positive at necropsy via fecal PCR performed at IDEXX BioAnalytics (Columbia, MO, USA).

### Mice

Mice bred in the three separate colonies harboring GM^Low^, GM^Low^+SFB, or GM^High^ were randomly assigned to one of four microbial exposures in a fully crossed study design, resulting in twelve treatment groups (Supplementary Figure S1). Randomization was performed using the RANDBETWEEN function in Microsoft Excel to assign mice from each colony to one of four microbial challenge groups. Microbial challenges include infection at weaning via gastric gavage with *Helicobacter hepaticus*, mouse hepatitis virus (MHV), co-infection with *H. hepaticus* and MHV, or sham inoculation. Two separate series of experiments were performed using different cohorts of mice, one sacrificed uniformly at 16 weeks of age and the other monitored for up to 52 weeks of age to determine effects of treatment on disease onset. For the 16-week experiment, each treatment group included 12 males (M) and 12 females (F) for a total of 288 mice (*n* = 24 mice [12M, 12F] per treatment group × 12 groups). For the 52-week study each treatment group included eight males and eight females for a total of 192 mice (*n* = 16 mice [8M, 8F] per treatment group × 12 groups). MHV-inoculated mice were housed separately from non-MHV inoculated mice due to required barrier conditions within our vivarium. MHV-negative mice (including colony mice) were housed within a barrier room at Discovery Ridge (DR), while MHV-positive mice were housed at the Laboratory Animal Center (LAC) within a barrier room, both accredited by the Association for Assessment and Accreditation of Laboratory Animal Care (AAALAC) International. All mice were in microisolator cages (positive pressure, 50 air changes per hour) on individually ventilated cage-racks (Thoren, Hazleton, PA, USA), filled with autoclaved compressed paper (Paperchip Brand Laboratory Animal Bedding, Shepherd Specialty Paper) with *ad libitum* access to autoclaved rodent chow (BreederDiet 5058 Purina) and water, under a 12:12 light/dark cycle. Cage change occurs every 2 weeks, and colonies with different gut microbiomes are changed on different days. The cages contained a nestlet for enrichment and mice were group-housed four per cage. The water provided to mice differed between facilities, MHV-negative and colony mice at DR received water via autoclaved Milli-Q Direct system (Merck KGaA, Darmstadt, Germany). While MHV-positive mice at LAC, for the duration of the study, received autoclaved, acidified water via an automated bottle filler which titrated the water with sulfuric acid (model 9WEF). These differences were purely due to accessibility of water source within each facility. Aside from experimental microbes, all mice were free of all adventitious rodent pathogens including MHV, Minute virus of mice (MVM), Mouse parvovirus (MPV), Mouse norovirus (MNV), Theiler's murine encephalomyelitis virus (TMEV), Epizootic diarrhea of infant mice (EDIM), Sendai virus, Pneumonia virus of mice (PVM), Reovirus Type 3 (REO3), Lymphocytic choriomeningitis virus (LCMV), Ectromelia virus (ECTV), Mouse adenovirus Type 1 and Type 2 (MAV1, MAV2), Murine polyomavirus (MPyV), Murine cytomegalovirus (MCMV), K virus, Lactate dehydrogenase-elevating virus (LDEV), Hantaan virus, and Moue thymic virus (MTV), all rodent *Helicobacter* spp., *Mycoplasma pulmonis*, beta-hemolytic streptococci (Groups A, B, C, G), *Citrobacter rodentium, Klebsiella oxytoca, Klebsiella pneumoniae, Streptococcus pneumoniae, Rodentibacter heylii, Rodentibacter pneumotropicus, Bordetella hinzii, Bordetella bronchiseptica, Filobacterium rodentium* (cilia-associated respiratory [CAR] bacillus), *Salmonella* spp.*, Clostridium piliforme, Cryptosporidium* spp. *Streptobacillus moniliformis, Corynebacterium kutscheri, Corynebacterium bovis, Encephalitozoon cuniculi, Pneumocystis murina*, fur mites, mesostigmatid mites, lice, *Spironucleus muris, Giardia muris*, large intestinal flagellates and amoeba, pinworms and tapeworms.

### Experimental inoculation

All mice were inoculated via gastric gavage at 3–4 weeks of age. Control mice were inoculated with 500 μL of autoclaved sterile Brucella broth as a sham inoculum. Mice selected for *H. hepaticus* and MHV+*H. hepaticus* treatment groups were administered 250 μL of *H. hepaticus* strain (MU-94) cultured in Brucella broth using previously described methods (Hillhouse et al., 2011). After *H. hepaticus* inoculation, mice in the MHV+*H. hepaticus* treatment group were then transferred to the LAC facility and inoculated with MHV the following day. MHV and MHV+*H. hepaticus* mice were inoculated via gastric gavage with 100 μL DMEM containing 2 × 10^5^ pfu of a confirmed disease-causing enterotropic strain of MHV isolated by and obtained from Greg Purdy at IDEXX BioAnalytics using previously described methods (Bauer et al., 2016). Feces were collected from all mice inoculated with *H. hepaticus* 2 weeks post-inoculation to confirm colonization via PCR. MHV infection was confirmed via serology.

### Blood glucose level monitoring

Starting at 10 weeks of age, blood glucose levels (BGL) were monitored twice weekly for the remainder of the experiment. Blood was collected from the saphenous vein during manual restraint, and BGL were measured using an AlphaTRAK 2 Blood Glucose Monitoring System Kit using code 91. Two separate devices were calibrated and used at each facility where mice were housed. For both studies, if a BGL measured 300 mg/dL or higher for two consecutive readings, the mouse was considered diabetic and was humanely euthanized via inhaled carbon dioxide, in accordance with the AVMA Guidelines for the Euthanasia of Animals: 2020 Edition depending on euthanasia date, and followed by cervical dislocation or pneumothorax as a secondary method. Approximately 0.4–0.6 mL of blood was collected immediately post-mortem via cardiocentesis and placed into a BD Microtainer^®^ gold top serum separator tube (SST). SSTs were then placed on an oscillator for 30 min, and then centrifuged at 10,000 × rpm for 5 min. Serum was collected into a 1.5 mL Eppendorf tube and placed in a −20 °C freezer. A subset of serum samples was submitted to IDEXX BioAnalytics for BGL measurement. These measurements were compared to BGL readings from the glucometer and demonstrated excellent agreement between methods of measuring BGL.

### Fecal sample collection

One to two freshly evacuated fecal pellets were collected from mice immediately prior to inoculation at 3–4 weeks of age, and again 2 weeks post-inoculation for *H. hepaticus*-infected mice. For sample collection, mice were placed individually in clean microisolator cage without bedding under a class II biological safety cabinet and allowed to defecate. Terminal feces were collected post-mortem by collecting the most distal fecal pellet in the rectum or distal colon. Fecal pellets were collected with sterile toothpicks and transferred into 2.0 mL round-bottom tubes containing a 0.5 cm-diameter stainless steel bead and immediately placed on ice. Cecal contents were collected from all mice post-mortem by creating a small incision at the tip of the cecum and squeezing cecal contents directly into a sterile 2.0 mL round-bottom tube containing a stainless steel bead. Whole pancreas was removed at necropsy and placed directly into a histological cassette and fixed in neutral-buffered 10% formalin for at least 24 h. Pancreas were submitted to IDEXX BioAnalytics for sample processing and hematoxylin and eosin (H&E) staining.

### Insulitis scoring

Sections of H&E-stained pancreas were scored by a trained veterinary pathologist (BO) blinded to the treatment groups. Each islet on an individual slide was scored for insulitis on a scale from 0 to 4 as followed: 0—No insulitis, 1—mild peri-insulitis occupying roughly 25% of the islet, 2—peri-insulitis occupying roughly 50% of the islet, 3—moderate intra-insulitis occupying roughly 75% of the islet, 4—severe insulitis occupying the majority of the islet. The overall score for each slide was calculated as the median of all individual islet scores for that slide (Jain et al., 2008).

### DNA extraction

Fecal and cecal DNA was extracted using QIAamp PowerFecal Pro kits (Qiagen, Venlo, Netherlands) according to the manufacturer's instructions, with the exception that samples were homogenized in 2.0 mL tubes containing a stainless steel ball bearing using a TissueLyser II (Qiagen) for 10 min at 30/s rather than utilizing the vortex adapter described in the manual protocol. Sample DNA was eluted in 100 μL of elution buffer (Qiagen) and DNA yields were quantified via fluorometry (Qubit 2.0, Invitrogen, Carlsbad, CA, USA) using quant-iT broad-range dsDNA reagent kits (Invitrogen).

### Confirmation of colonization or infection

Two-week post-inoculation with *H. hepaticus*, fecal samples were collected from *H. hepaticus-* and MHV+*H. hepaticus*-inoculated mice. Fecal DNA was extracted, and PCR was performed to confirm the presence of *H. hepaticus* in feces. For the 16-week study, colonization was confirmed in our laboratory by PCR using a previously established protocol with primers specific for *H. hepaticus* (Drazenovich et al., 2002). For the 52-week study, fecal DNA was submitted to IDEXX BioAnalytics for PCR confirmation of presence of *H. hepaticus* using the same primers and protocol. Mice that were found to be negative for *H. hepaticus* via PCR post-inoculation were removed from study.

Colonization of MHV was confirmed via the serum of MHV- and MHV+*H. hepaticus-*treated mice, collected at euthanasia following both the 52- and 16-week studies. A single serum sample was chosen at random to represent each cage, and a cage was considered positive if at least one mouse per cage tested positive for MHV. These randomly selected samples were then submitted to IDEXX BioAnalytics for serologic analysis. For mice not inoculated with MHV, which were housed at a separate facility devoid of MHV, sentinel mice were utilized to confirm MHV-negative status.

Colonization of SFB was confirmed via PCR analysis of pooled DNA from feces collected at the pre-treatment timepoint (i.e., 3–4 weeks of age) and at necropsy for both studies. Samples were pooled by cage after DNA extraction, where samples DNA concentration was diluted to equally represent each animal in the cage at a concentration of 20 ng/μL. Pooled samples were then submitted to the IDEXX BioAnalytics for PCR analysis, and all mice regardless of background GM were tested.

### 16S rRNA library preparation and sequencing

Extracted mouse fecal and cecal DNA were processed at the University of Missouri Genomics Technology Core facility. Bacterial 16S rRNA amplicons were constructed by amplifying the V4 region of the 16S rRNA gene with universal primers (U515F/806R) flanked by Illumina standard adapter sequences using previously described methods (Walters et al., 2011; Caporaso et al., 2011). Oligonucleotide sequences are stored for reference at proBase (Loy et al., 2007). Forward and reverse dual-indexed primers were using in all reactions. PCR reactions were performed at a volume of 50 μL and contained 100 ng metagenomic DNA, primers (0.2 μM each), dNTPS (200 μM each), and Phusion high fidelity DNA polymerase (1U, Thermo Fisher, Waltham, MA). Parameters for amplification were 98 °C^(3min)^ + [98 °C^(15s)^ + 50 °C^(30s)^ + 72 °C^(30s)^] × 25 cycles + 72 °C^(7min)^. Amplicon pools at 5 μL/reaction were combined by thorough mixing and subsequently purified by adding Axygen Axyprep MagPCR clean-up beads to an equal volume of 50 μL and incubated at room temperature for 15 min. After purification, products received multiple washes of 80% ethanal and the dried pellet was resuspended in 32.5 μL EB buffer (Qiagen), incubated at room temperature for 2 min, and then placed on a magnetic stand for 5 min. The final amplicon pool was then evaluated using the Advanced Analytical Fragment Analyzer automated electrophoresis system, quantified using quant-iT HS dsDNA reagent kits, and finally diluted according to Illumina's standard sequencing protocol as 2 × 250 bp paired-end reads on the MiSeq instrument.

### Bioinformatics

Fecal and cecal DNA sequences were assembled and annotated at the University of Missouri Bioinformatics and Analytics Core facility. Cutadapt (Martin, 2011) was used to removed primers, and an error-rate of 0.1 was allowed. The QIIME2 (Bolyen et al., 2019) DADA2 (Callahan et al., 2016) plugin (version 1.18.0) was used to denoise, de-replicate, and count ASVs (amplicon sequence variants). Python version 3.8.10 and Biom version 2.1.10 were used in QIIME2. Taxonomies were assigned to final sequences using the SILVA v138 (Pruesse et al., 2007) database, using the classify-sklearn procedure (Kaehler et al., 2019). For downstream analysis, data from both studies were rarefied to a uniform depth of 15,954 sequence reads per sample. Rarefied 16S rRNA sequencing data obtained from cecal contents of NOD mice from the 16-week old cohort were utilized for prediction of metagenome function using the PICRUSt2 (Douglas et al., 2020) version 2.5.0 algorithm. Data was separated by GM for each sex and analyzed independently. Phylogenic placement of reads was handled by gappa (Czech et al., 2020) version 0.8.0 with the alternative placement option SEPP (Mirarab et al., 2011) version 4.5.1. The R library Castor (Louca and Doebeli, 2018) version 1.7.3 functions were utilized for hidden state phylogenetic predictions, and MinPath (Ye and Doak, 2009) version 1.4.0 was used for pathway inference.

### Statistical analysis

For analysis of T1D onset in the 52-week study, Kaplan–Meier Log-rank analyses were utilized to determine statistical differences between survival of treatment groups harboring each GM composition within male and female mice. Then, to determine the overall effect of sex, GM, and treatment on hyperglycemic onset, a Cox proportional hazard analysis was utilized. All statistics for T1D onset were generated using SigmaPlot 13.0.0, and graphs were generated using the R package ggplot2 (Wickham, 2016) version 3.3.6. Statistics for insulitis scores of mice from the 16-week cohort were calculated using a three-factor analysis of variance (ANOVA) on the factors sex, GM, and treatment. A three-factor ANOVA was generated using R package rstatix (Kassambara, 2021) version 0.7.0. Holm-Sidak pairwise comparisons for treatment groups were generated using the R package emmeans (Lenth, 2022) version 1.8.0. Bray-Curtis distribution matrices were generated first for male and female 16-week NOD mouse from pre-treatment fecal samples and terminal cecal content 16S rRNA sequencing data. One-way Permutational Multivariate Analysis of Variance (PERMANOVA) tests were used to determine significance of GM and collection timepoint on beta diversity, and Bray-Curtis *post hoc* analyses with Bonferroni adjustments were utilized to determine pairwise differences. Next, Bray-Curtis distributions and PERMANOVA for treatment differences within each GM for male and female mice were utilized following the same statistical procedure. One-way PERMANOVA was computed using the R package vegan (Oksanen et al., 2022) version 2.6.2, and pairwise comparisons were generated with the R package ecole version 09-2021 (Smith, 2021). Principal coordinate analysis (PCoA) graphs generated from Bray-Curtis distributions with the cailliez adjustment method via R package ape (Paradis and Schliep, 2018) version 5.6.2. PICRUSt2 metagenome pathway predictions were analyzed for significant differences between treatment groups within each GM and sex by utilizing a nested ANOVA on ranks test followed by a Dunn's *post hoc* with Benjamini–Hochberg corrections for pairwise comparison of treatments. The R package stats (R Core Team, 2022) version 4.2.1 was used to calculate these statistics.

## Results

### Disease modifying organisms (DMOs) have modest *e*ffect on insulitis during early stage of disease

**Figure 1:**
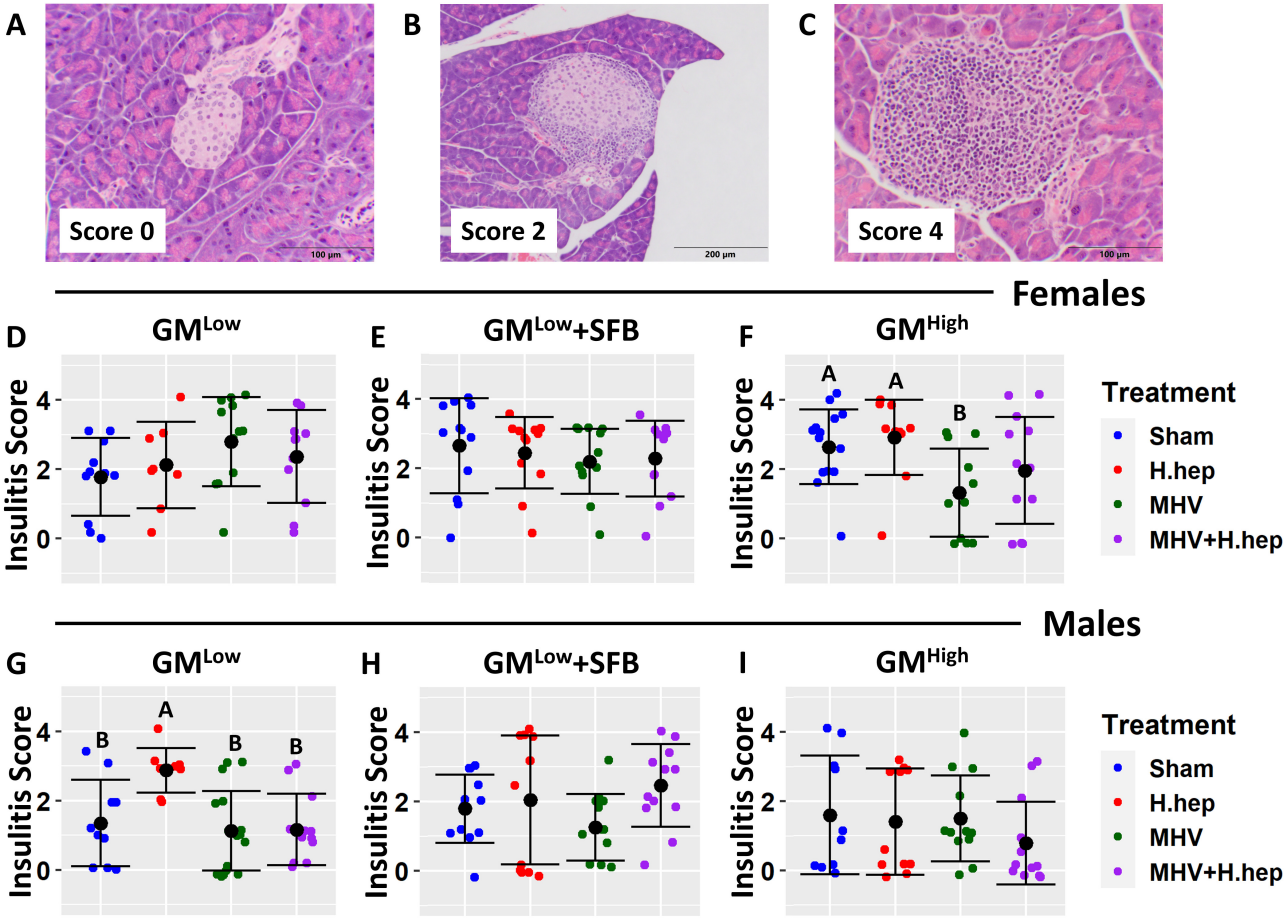
Pathobiont infection affects insulitis during early stages of disease. Representative histological images of pancreatic islets showing examples of individual islets receiving a score 0, indicating no lymphocyte infiltration **(A)**, score 2, indicating roughly 50% of the islet is occupied by infiltrating lymphocytes **(B)**, and score 4, indicating almost total infiltration of the islet space **(C)**; examples of intermediate scores 1 and 3 not shown. Dot plots showing pathobiont-associated differences in median insulitis scores of female mice colonized with GM^Low^ **(D)**, GM^Low^+SFB **(E)**, or GM^High^ **(F)**, or male mice colonized with the same resident microbiomes **(G–I)**. Black dots and error bars represent group means and standard deviation. Data were analyzed using a three-factor ANOVA followed by pairwise comparisons using the Holm-Sidak method. Significant (*p* < 0.05) differences between treatment groups within each GM are designated by different letters. Legend at lower right, H.hep, *Helicobacter hepaticus*; MHV, mouse hepatitis virus; MHV+H.hep, co-infection with both agents.

To determine whether our variables of interest (Supplementary Figure S1) affect early stages of disease development prior to hyperglycemia, we assessed insulitis severity in a cohort of NOD mice at 16 weeks of age via histological scoring of lymphocytic infiltration in individual islets. Blood glucose levels were assessed twice weekly beginning at 10 weeks of age, and none of the mice in any group demonstrated hyperglycemia (i.e., BGL >300 mg/dL) at any time prior to 16 weeks. A five-point ordinal scale was used wherein a score of 0 indicates no insulitis (Figure 1A), a score of 2 indicates approximately 50% of the islet is occupied by lymphocytes (Figure 1B) and a score of 4 indicates severe insulitis with minimal to no visible beta cells (Figure 1C). In agreement with the well-recognized sex bias in this model, three-factor ANOVA detected a significant main effect of sex (*F* = 24.1, *p* < 0.001) reflecting more severe insulitis in female mice, and a significant main effect of treatment (*p* = 0.046, *F* = 2.7) and interaction between all three factors (*p* = 0.005, *F* = 3.2). *Post hoc* tests indicated that mice infected with MHV, regardless of GM or sex, had lower insulitis scores than *H. hepaticus* (H.hep)-infected mice (*t* = 2.8, *p* = 0.031). Considering the significant interaction of all three factors, we next tested the effect of DMO treatments within female or male mice of each GM background separately. Within female mice, there was no significant difference between treatment groups (i.e., MHV, H.hep, MHV+H.hep, or control) in mice colonized with GM^Low^ (Figure 1D) or GM^Low^+SFB (Figure 1E). Within GM^High^-colonized females (Figure 1F), MHV-infected mice had significantly lower insulitis scores than either control (*t* = 2.7, *p* = 0.043) or H.hep-infected mice (*t* = 3.1, *p* = 0.014). Within males, there was a significant effect of treatment in GM^Low^-colonized mice (Figure 1G), where H.hep-infected mice developed more severe insulitis than control mice (*t* = 2.6, *p* = 0.041), MHV-infected mice (*t* = 3.3, *p* = 0.001), and MHV+H.hep co-infected mice (*t* = 3.0, *p* = 0.014). No treatment-associated differences were detected in male mice colonized with GM^Low^+SFB (Figure 1H) or GM^High^ (Figure 1I). Collectively, these data suggested that DMOs selectively influence insulitis depending on the sex and resident microbiome, and that H.hep and MHV are associated with increased or decreased insulitis, respectively, during early stages of disease.

### DMO exposure, particularly *H. hepaticus*, affects composition of the microbiome

**Figure 2:**
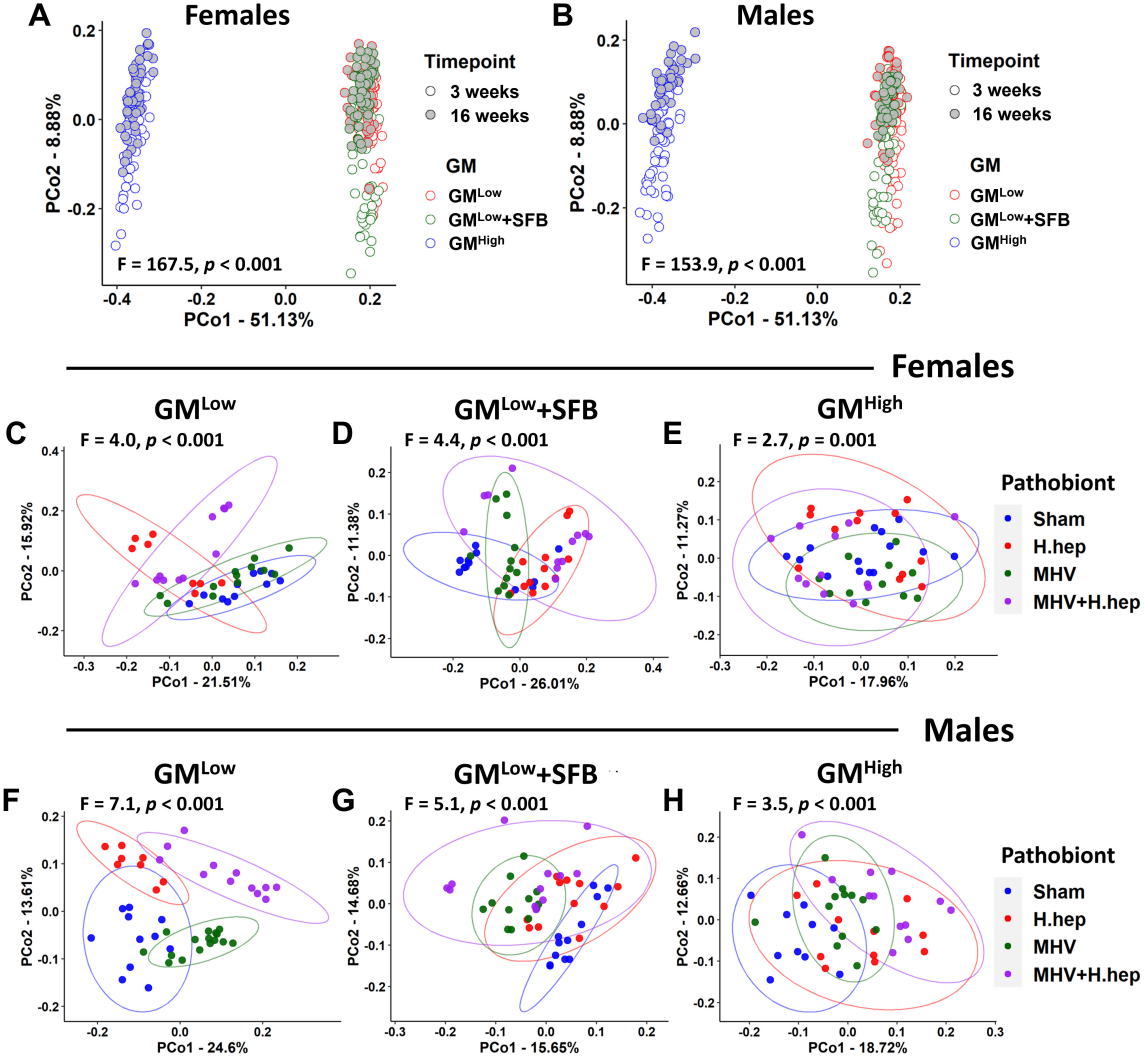
Helicobacter induces significant changes in composition of the microbiome. Principal coordinate analysis (PCoA) plots based on Bray-Curtis dissimilarities in the fecal microbiome of female **(A)** and male **(B)** NOD mice at 3 weeks (open circles) and 16 weeks (filled circles) of age, colonized with GM^Low^, GM^Low^+SFB, or GM^High^; legend at right, *p* and *F* values indicate GM-associated effects (PERMANOVA). PCoA plots showing treatment-associated effects in beta-diversity at 16 weeks of age in female **(C–E)** and male **(F–H)** mice colonized with GM^Low^ **(C, F)**, GM^Low^+SFB **(D, G)**, or GM^High^ **(E, H)**. Legend at lower right, H.hep = *Helicobacter hepaticus*, MHV = mouse hepatitis virus, MHV+H.hep = co-infection with both agents.

To confirm the expected difference between GM^Low^ and GM^High^, and to determine whether SFB, H.hep, or MHV were associated with significant changes in the gut microbiome, fecal samples collected at weaning and cecal contents collected at necropsy from the 16-week cohort of mice were used as a source of DNA for 16S rRNA amplicon sequencing. Principal coordinate analysis (PCoA) and permutational multivariate analysis of variance (PERMANOVA) were used to visualize and test for differences in beta-diversity, respectively. Separate PCoA of samples from female (Figure 2A) and male (Figure 2B) mice revealed a stark separation of GM^High^ samples from the other two GMs along PC1 in both sexes. Samples from GM^Low^- and GM^Low^+SFB-colonized mice clustered together, and similar movement along PC2 occurred in all groups between three and 16 weeks. As expected based on sample ordination, two-factor PERMANOVA revealed comparable effects of GM and timepoint in females (GM, *F* = 167.5, *p* < 0.001; timepoint, *F* = 14.0, *p* < 0.001) and males (GM, *F* = 153.9, *p* < 0.001; timepoint, *F* = 16.8, *p* < 0.001).

To circumvent those strong effects, we stratified data by GM and 16-week timepoint to assess treatment-associated effects on beta-diversity. A compelling pattern of *H. hepaticus*-associated effects was observed in female mice colonized with GM^Low^ (Figure 2C), GM^Low^+SFB, (Figure 2D), and GM^High^ (Figure 2E). Specifically, PERMANOVA revealed a significant effect of treatment in all three GMs (GM^Low^, *F* = 4.0, *p* < 0.001; GM^Low^ +SFB, *F* = 4.4, *p* < 0.001; GM^High^, *F* = 2.7, *p* = 0.001). Pairwise comparisons detected significant differences between control mice and *H. hepaticus*-infected or *H. hepaticus*/MHV co-infected mice, but not MHV-infected mice, in both GM^Low^-colonized (H.hep, *F* = 4.8, *p* = 0.006; MHV, *F* = 2.3, *p* = 0.07; MHV+H.hep, *F* = 5.5, *p* = 0.018) and GM^High^-colonized (H.hep, *F* = 3.6, *p* = 0.018; MHV, *F* = 2.0, *p* = 0.108; MHV+H.hep, *F* = 3.3, *p* = 0.006) mice. In GM^Low^+SFB-colonized females (Figure 2D), the microbiome of MHV-infected mice was significantly different from controls, albeit to a lesser degree than either *H. hepaticus*-infected group (H.hep, *F* = 6.1, *p* = 0.006; MHV, *F* = 2.9, *p* = 0.030; MHV+H.hep, *F* = 5.2, *p* = 0.006). Effects of treatment assessed similarly in male mice colonized with GM^Low^ (Figure 2F), GM^Low^+SFB, (Figure 2G), and GM^High^ (Figure 2H) revealed significant effects of treatment in all three GMs (GM^Low^, *F* = 7.1, *p* < 0.001; GM^Low^+SFB, *F* = 5.1, *p* < 0.001; and GM^High^ *F* = 3.5, *p* < 0.001). In male mice, PERMANOVA detected significant differences in beta-diversity between control mice and all treatment groups (including MHV-infected mice), in all three GMs. However, as in females, the effects of *H. hepaticus* were greater than MHV, based on sample ordination and results of pairwise comparisons during PERMANOVA.

### Colonization with *H. hepaticus* induces inferred changes in metabolic function

**Figure 3:**
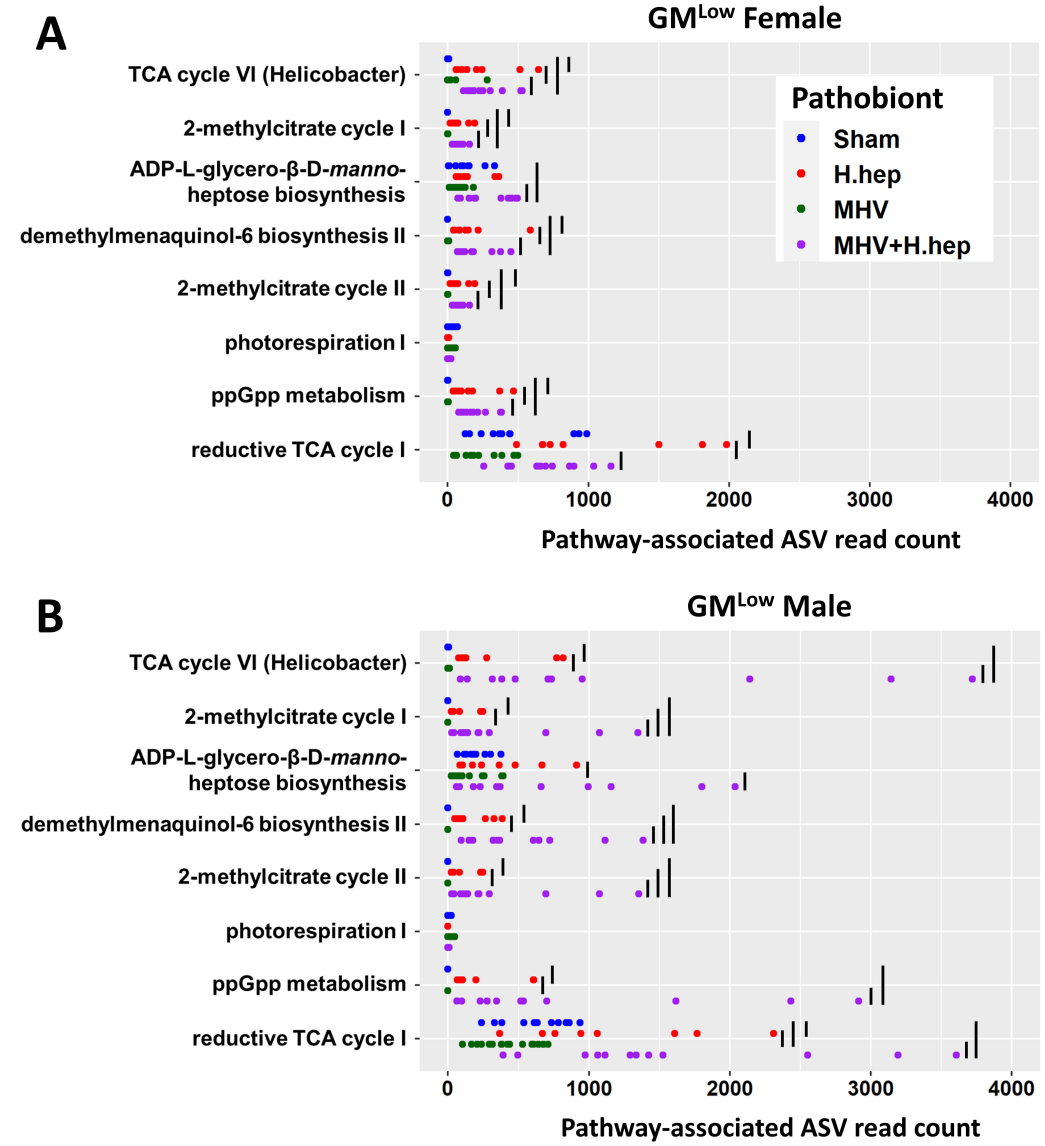
Helicobacter-induced changes in beta-diversity predicted to affect sugar metabolism. Dot plots showing inferred metagenomic content of metabolic pathways identified as differentially abundant across all groups. Representative data from GM^Low^-colonized female **(A)** and male **(B)** mice are shown. Bars indicate significant differences between treatment groups within each GM and sex based on a nested ANOVA on ranks test followed by a Dunn's *post hoc* and correction for multiple tests using the method of Benjamini and Hochberg. Legend at lower right, H.hep, *Helicobacter hepaticus*, MHV, mouse hepatitis virus; MHV+H.hep, co-infection with both agents.

To determine if these differences in phylogenetic composition were associated with changes in imputed function (predicted based on taxonomic composition), we utilized the PICRUSt2 pipeline (Douglas et al., 2020). Data were tested for treatment-associated differences within each GM, in both female and male mice. Significant differences in predicted pathway-associated read counts were determined for each analysis, and consistently enriched pathways across all analyses were identified, including TCA cycle IV (Helicobacter), 2-methylcitrate cycle I, ADP-L-glycero-β-D-*manno*-heptose biosynthesis, demethylmenaquinol-6 biosynthesis II, 2-methycitrate cycle II, photorespiration I, ppGpp metabolism, and reductive TCA cycle I. Treatment-associated effects on predicted functional pathways in GM^Low^-colonized female (Figure 3A) and male (Figure 3B) mice demonstrate a consistent pattern of *H. hepaticus*-dominated effects, repeated in GM^Low^+SFB (Supplementary Figures S2A, B) and GM^High^ (Supplementary Figures S2C, D) mice. The presence of MHV, alone or in combination with *H. hepaticus*, had minimal effect on predicted function. These data suggest that inoculation with *H. hepaticus* at weaning may be associated with conserved changes in function of the resident microbiome, characterized by changes in energy harvest facilitated by the TCA cycle.

### Disease onset is influenced by interactions between microbiome and DMOs

**Figure 4:**
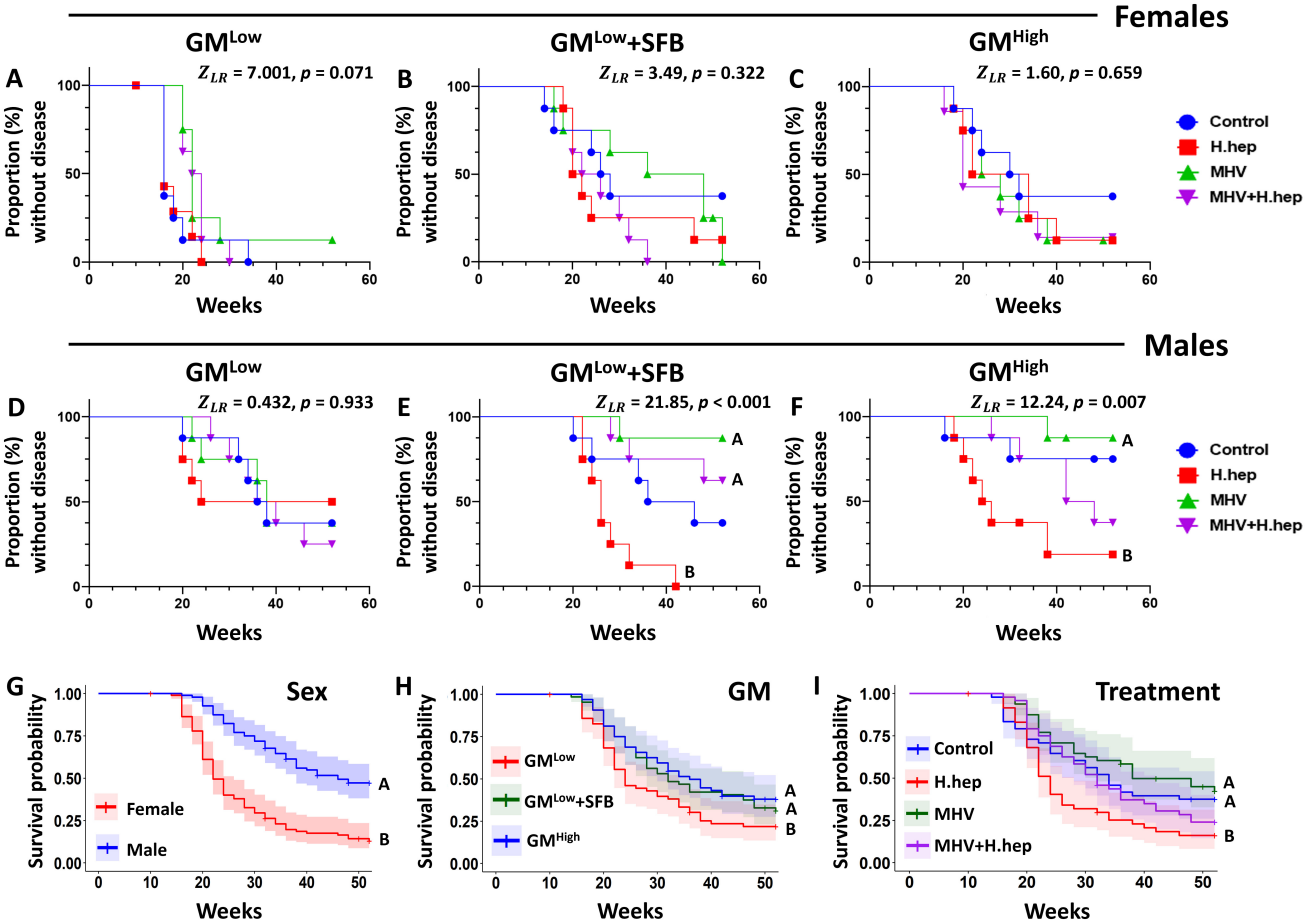
Disease onset is driven by interactions between microbiome and pathobionts. Kaplan-Meier survival curves for incidence of type 1 diabetes, defined as two consecutive blood glucose level readings greater than 300 mg/dL, over the course of 52 weeks in female mice colonized with GM^Low^ **(A)**, GM^Low^+SFB **(B)**, or GM^High^ **(C)**, or male mice colonized with the same resident microbiomes **(D–F)**. Legend at right, H.hep, *Helicobacter hepaticus*, MHV, mouse hepatitis virus; MHV+H.hep, co-infection with both agents. Data were analyzed using Log-rank tests within each group, and the Holm-Sidak method for *post hoc* pairwise comparisons. Significant (*p* < 0.05) differences between treatment groups within each GM are designated by different letters. Kaplan–Meier curves representing the univariate Cox proportional hazards analysis of survival probability associated with each factor including sex **(G)**, gut microbiome (GM) (**H**), and treatment **(I)**. Key for each comparison overlaid on graph. Shading represents 95% confidence interval. Significant (*p* < 0.05) differences between groups within each comparison are designated by different letters.

Lastly, we performed a 52-week study to determine if the gut microbiome or any of the experimental inoculations were capable of altering the timing of disease onset. A fully crossed study design including all three background GMs and four experimental inoculation groups per GM, was replicated in males and females (*n* = 8 mice/sex/GM/treatment for *N* = 288 in total). Kaplan-Meier survival curves and log-rank tests were utilized to determine if there was an overall difference between treatment groups within each GM and sex. Log-rank analysis found no significant differences between treatment groups within female mice colonized with GM^Low^ (Figure 4A), GM^Low^+SFB (Figure 4B), or GM^High^ (Figure 4C), or male mice colonized with GM^Low^ (Figure 4D). Log-rank analysis did find an overall significant difference in disease incidence between treatment groups in male mice colonized with GM^Low^+SFB (Figure 4E), and *post hoc* analysis revealed significantly greater disease incidence in *H. hepaticus*-infected mice compared to mice infected with MHV (*t* = 13.9, *p* < 0.001) or co-infected with MHV and *H. hepaticus* (*t* = 11.3, *p* = 0.004). Similarly, log-rank analysis detected treatment-associated differences in male mice colonized with GM^High^, (Figure 4F) and *post hoc* analysis found that *H. hepaticus*-infected mice had higher disease incidence than MHV-infected mice (*t* = 8.2, *p* = 0.037).

To isolate the effect of each factor on disease incidence, a Cox proportional hazards regression analysis was used (Supplementary Table S1). As expected, female mice had a significantly higher Wald (*W*) Chi-squared statistic, and therefore a higher likelihood of disease than male mice [*W* = 43.2, hazard ratio (HR) = 0.29, χ^2^ = 43.2, *p* < 0.001; Figure 4G). Similarly, mice harboring GM^Low^ had a greater likelihood of disease than mice colonized with either GM^Low^+SFB (*W* = 8.0, HR = 0.55, χ^2^ = 8.0, *p* = 0.005) or GM^High^, (*W* = 12.4, HR = 0.46, χ^2^ = 12.4, *p* < 0.001; Figure 4H), and *H. hepaticus*-infected mice had a higher likelihood of disease than both control (*W* = 5.3, HR = 0.57, χ^2^ = 5.3, *p* = 0.021) mice and MHV-infected (*W* = 12.2, HR = 0.41, χ^2^ = 12.2, *p* < 0.001) mice (Figure 4I).

## Discussion

These findings have several implications related to the NOD mouse model, humans affected with T1D, and the specific mechanisms driving autoimmune disease in both. To test the influence of these factors on disease incidence in the NOD mouse model, we used different supplier-origin microbiomes previously shown to affect disease severity in numerous immune-mediated disease models (Hart et al., 2017; Moskowitz et al., 2019; Velazquez et al., 2019; Stough et al., 2016; Villarino et al., 2016), and three provocateurs or disease-modifying organisms (DMOs) purportedly capable of mitigating or exacerbating disease severity of mouse models depending on the disease pathogenesis. The three DMOs used here (SFB, *H. hepaticus*, and MHV) were selected based on their typically subclinical nature, high prevalence (Albers et al., 2023), prior associations with disease incidence, and the presence of cognate organisms in humans.

In terms of experimental reproducibility, these data highlight the influence of the background GM and all three DMOs and provide a potential explanation for phenotypic variability between labs or over time. These data also suggest a means for investigators to enhance or decrease disease severity in this model, via selection of the optimal microbial exposures. Prior studies documented greater disease severity in NOD mice infected with *H. hepaticus* (De Riva et al., 2017), and reduced disease severity in mice colonized with SFB (Fahey et al., 2017; Kriegel et al., 2011; Rouland et al., 2022) or infected with MHV (Wilberz et al., 1991). None of those reports, however, used prospective experimental infection. Rather, each reported differential disease incidence in colonies wherein each microbe was found to be present or absent. The present data, generated in two independent but identically designed prospective, fully crossed studies, support the conclusions of those studies. These findings also suggest the NOD mouse model can be useful in research investigating mechanisms whereby gut microbes influence the development of spontaneous autoimmune disease. The NOD mouse model was selected for these studies based on its anecdotally high degree of variability in disease incidence between labs, and the present data confirm that early-stage insulitis and disease onset are both highly variable and influenced by complex interactions between multiple specific DMOs and the native gut microbiome.

Our results also suggest a possible interaction effect between sex and treatment group on disease incidence. As anticipated, we found a significant effect of sex, with higher overall incidence in female mice. The effects of DMOs on disease incidence however were more apparent in male mice, evidenced by marked differences in disease incidence between groups (e.g., MHV-infected vs. *H. hepaticus*-infected), particularly in those with GM^Low^+SFB and GM^High^ microbiomes. It may be that the autoimmune response in females is of sufficient strength to obscure the effects of these microbes. That said, the protective effect of GM^Low^+SFB and GM^High^ microbiomes is evident in female mice, as all GM^Low^ female groups except MHV-infected experienced 100% incidence by 35 weeks of age, while only two GM^Low^+SFB groups reached 100% mortality (at 36 and 52 weeks) and none of the GM^High^ groups did.

While the mechanisms underlying the effects of these microbes are unclear, it is reasonable to speculate that the microbes are somehow influencing the immune responses responsible for insulitis. MHV is usually subclinical in both immunocompetent laboratory mice as well as wild mice (Barthold and Smith, 1990; Körner et al., 2020; Parker et al., 2009). Broadly speaking, there are virulent polytropic strains of MHV and less virulent enterotropic strains (Homberger, 1997) such as the one used in the current study. Depending on the strain, MHV can induce either depletion or expansion of T cells (Kyuwa et al., 1996; Khanolkar et al., 2009; Stevenson and Doherty, 1998; Godfraind et al., 1995; Lee et al., 1994). Subclinical adaptive immune responses to MHV can influence other model phenotypes, including enhanced protection from unrelated infectious agents (Fallon et al., 1991) and, alternatively, exacerbated disease in autoimmune conditions (Aparicio et al., 2011; Musaji et al., 2004, 2005). Our data are in accord with previous work suggesting a protective effect of MHV in the NOD model (Wilberz et al., 1991).

Similarly, SFB, *H. hepaticus*, and many other enterohepatic *Helicobacter* spp. are endemic in wild mice as part of the native microbiome. Each of these microbes induce the development or maintenance of different components of the adaptive immune system. Experimental inoculation of naïve mice with *H. hepaticus* induces a potent CD4^+^ Th1 cell response (Morrison et al., 2013; Whary et al., 1998; Kullberg et al., 2003), while SFB are critical to the development and maintenance of CD4^+^ Th17 cells (Goto et al., 2014; Ivanov et al., 2009). Disease pathogenesis in NOD mice is dependent on both CD8^+^ and CD4^+^ cell activity. While CD4^+^ Th1, Th2, and Th17 effector cells can all potentiate disease severity in NOD mice, regulatory Th17 cells confer a protective effect (Trembleau et al., 1995; Pakala et al., 1997; Singh et al., 2013). We speculate that the observed effects of DMOs and background GMs reflect the combined balance of immune cell activity during disease development.

Our findings also suggest an alternative means by which these DMOs may affect disease onset. *H. hepaticus* in particular was associated with changes in the composition of the background GM, Experimental inoculation with *H. hepaticus* induces acute and lasting changes in the composition of the GM in other models (Wolfe et al., 2020; Swennes et al., 2014; Kuehl et al., 2005), making it difficult to discern direct and indirect effects of *Helicobacter* spp. on model phenotypes. Here, the changes in taxonomy induced by *H. hepaticus* were associated with a highly consistent set of predicted changes in metagenomic gene content. The eight pathways consistently affected by *H. hepaticus* infection were related to TCA cycle regulation, degradation of propionate, and biosynthesis of vitamin K2 and related metabolites. Notably, longitudinal analysis of the serum metabolome in NOD and C57BL/6 mice suggests decreased TCA cycle activity in NOD mice during the pre-diabetic state (Madsen et al., 2012). Thus, infection with *H. hepaticus* may contribute to an existing change in energy metabolism associated with early stages of disease. This effect may not be specific to NOD mice as *H. hepaticus*-infected Rag2^−^/^−^ mice also experience changes in the gut metabolome including reduced TCA cycle activity and fatty acid synthesis alongside exacerbation of disease (Lu et al., 2012).

Regarding the translatability of these findings to the human condition, it is noteworthy that cognate species of these DMOs are present in the human population. While DNA specific for SFB is rarely detected in adult feces, analysis of fecal samples from infants, toddlers, and children shows that colonization with SFB in humans follows the same dynamic observed in rodents, with high levels of fecal DNA in early life followed by a reduction to the point of being undetectable by adulthood (Yin et al., 2013; Jiang et al., 2001). SFB selectively colonize the ileal mucosa, a site rich in mucosa-associated lymphoid tissue, and the influence of SFB on immune system development in humans appears to parallel that observed experimentally in rodents (Ivanov et al., 2009; Chen B. et al., 2018). Similarly, there are numerous *Helicobacter* spp. that can colonize the human gastrointestinal tract, including *H. pylori* and numerous non-*H. pylori Helicobacter* (NHPH) species that are increasingly associated with human disease beyond gastric ulcers (Fox, 2002; Rossi and Hanninen, 2012). Lastly, MHV is a coronavirus, related in many ways to the human coronavirus associated with the COVID-19 pandemic (Körner et al., 2020; Grabherr et al., 2021). Whether in rodents or humans, these organisms induce immune responses that may be clinically silent. Considering the multifactorial nature of T1D however, these and other resident DMOs (Cani et al., 2022; Collado et al., 2007; Derrien et al., 2008; Herp et al., 2021) may need to be considered in the current era of precision medicine.

There are certain limitations to the study that affect the interpretability of our findings. First, mice in each study were housed in two different facilities on the University of Missouri (MU) campus, depending on their MHV status. All mice used in both studies were generated in the same high-biosecurity vivarium, and mice were transported to a separate facility for housing post-MHV infection. While diet and husbandry at each facility was matched as closely as possible, one unavoidable variable was water sterilization method. Recognizing the potential effect of that or other unrecognized factors on GM composition (Bidot et al., 2018), no differences in beta-diversity or relative abundance of features were detected between mice based on these factors. The effect of acidified drinking water on disease incidence in NOD mice has been investigated previously, with conflicting results. Two initial studies published within months of each other found that acidified water either exacerbated (Sofi et al., 2014) or mitigated (Wolf et al., 2014) disease incidence in NOD mice, and both reported changes in the microbiome of mice consuming acidified water. Subsequent attempts to reconcile these findings by others have failed to detect any effect at all (Zhao and Tarbell, 2014; Jayaraman and Jayaraman, 2018), leading to speculation that these discrepancies may be explained by differences in the baseline microbiome of mice in each study (Zhao and Tarbell, 2014). Thus, while our findings related to MHV are in agreement with previous retrospective analysis (Wilberz et al., 1991) and the effects of acidified water on disease incidence of NOD mice are questionable, we cannot rule out the potential effect of acidified water or other unrecognized variables between facilities.

Second, no outcome measures were included to characterize the innate and adaptive immune response in each group. While additional data related to T and B cell activity and cytokine levels in the islets or peripheral circulation could help indicate mechanisms explaining the observed differences, we focused on disease onset and rely on the abundant literature (Goto et al., 2014; Ivanov et al., 2009; Lécuyer et al., 2014; Myles et al., 2007; Whary et al., 1998; Khanolkar et al., 2009; Talham et al., 1999; Wang et al., 2019; Myles et al., 2003; Jergens et al., 2006, 2007; Homberger and Barthold, 1992; Homberger et al., 1992) regarding immune responses induced by the microbial challenges used herein to speculate on potential explanatory mechanisms. The experimental objectives of this project were to prospectively validate previous retrospective studies that reported effects of each DMO (SFB, *H. hepaticus*, and MHV) on disease incidence in NOD mice, and to test for interactions among those DMOs and different background microbiome compositions. Conceptually, the objective was to identify microbiological sources of variability in the NOD model, as part of the greater effort to enhance research reproducibility between and within laboratories.

Additionally, we note that PICRUSt2 (Douglas et al., 2020) analyses do not reveal true metagenomic content, but rather the predicted metagenomic content based on taxonomic composition. Analysis of the fecal and serum metabolome via gas or liquid chromatography-mass spectrometry would be needed to validate differences in the metabolic intermediaries identified here.

## Conclusion

Collectively, the data presented here demonstrate the influence of three DMOs on disease incidence in the NOD mouse model. Specifically, *H. hepaticus* was associated with increased disease incidence while SFB and MHV each conferred a protective effect. Similarly, the richer native GM originating from Inotiv mice was associated with lower disease incidence than the less rich GM originating from The Jackson Laboratory, possibly due to its inclusion of SFB. These data are relevant to the experimental reproducibility of the NOD mouse model and highlight the need for researchers using NOD mice to test for these organisms in their colonies, and report the infection status in all publications. Ideally, samples would be obtained from weaning-age mice from the colony rather than sentinels and also include assessments of the microbiome. These data also highlight the potential utility of NOD mice in research investigating host-microbe interactions during autoimmune disease. Moreover, these and other DMOs need to be considered in the context of human T1D, not as direct causative factors, but as positive and negative risk factors that can be manipulated to reduce disease risk.

## Data Availability

All 16S rRNA amplicon sequencing data supporting the current study are freely available at the National Center for Biotechnology Information (NCBI) Sequence Read Archive (SRA) under BioProject PRJNA1046028.

## Supplementary material

Supplementary Figure S1 Schematic showing the experimental groups and primary outcomes. All experimental mice were offspring from one of three separate genetically identical colonies of non-obese diabetic (NOD/ShiLtJ) mice with low-richness gut microbiome (GMLow), GMLow supplemented with segmented filamentous bacteria (SFB), or high-richness GM (GMHigh) which naturally contains SFB. At weaning (21 d), mice from each colony were randomized into one of four treatments, sham inoculation (control), *Helicobacter hepaticus*, enterotropic Mouse Hepatitis Virus (MHV), or *H. hepaticus* and MHV. Mice in both cohorts were monitored twice weekly beginning at 10 weeks of age for hyperglycemia, defined as two consecutive blood glucose readings >300 mg/dL. Mice from Cohort 1 (*n* = 16 [8 males, 8 females] per group Ã-−12 groups = 192 mice) were uniformly sacrificed at 16 weeks of age to investigate early-stage disease via insulitis and changes in the GM. Mice from Cohort 2 (*n* = 24 [12 males, 12 females] per group Ã-−12 groups = 288 mice) were sacrificed at the confirmation of hyperglycemia or 52 weeks of age to investigate effects on disease onset.

Supplementary Figure S2 Helicobacter-induced changes in beta-diversity predicted to affect sugar metabolism. Dot plots showing imputed metagenomic content of metabolic pathways identified as differentially abundant across all groups. Representative data from GMLow+SFB-colonized female (A) and male (B) mice, and GMHigh-colonized female (C) and male (D) mice, are shown.

Supplementary Table S1 Results of Cox proportional hazards regression analysis for each comp.

## References

[B1] Alam C. Bittoun E. Bhagwat D. Valkonen S. Saari A. Jaakkola U. . (2011). Effects of a germ-free environment on gut immune regulation and diabetes progression in non-obese diabetic (NOD) mice. Diabetologia 54, 1398–1406. doi: 10.1007/s00125-011-2097-5 21380595

[B2] Albers T. M. Henderson K. S. Mulder G. B. Shek W. R. (2023). Pathogen prevalence estimates and diagnostic methodology trends in laboratory mice and rats from 2003 to 2020. J Am. Assoc. Lab. Anim. Sci. 62, 229–242. doi: 10.30802/AALAS-JAALAS-22-000097 37127407 PMC10230541

[B3] Aparicio J. L. Pena C. Retegui L. A. (2011). Autoimmune hepatitis-like disease in C57BL/6 mice infected with mouse hepatitis virus A59. Int. Immunopharmacol. 11, 1591–1598. doi: 10.1016/j.intimp.2011.05.020 21635973 PMC7106302

[B4] Bao M. Yang Y. Jun H. S. Yoon J. W. (2002). Molecular mechanisms for gender differences in susceptibility to T cell-mediated autoimmune diabetes in nonobese diabetic mice. J. Immunol. 168, 5369–5375. doi: 10.4049/jimmunol.168.10.5369 11994496

[B5] Barthold S. W. Smith A. L. (1990). Duration of mouse hepatitis virus infection: studies in immunocompetent and chemically immunosuppressed mice. Lab. Anim. Sci. 40, 133–137. 2157090

[B6] Bauer B. A. Besch-Williford C. Livingston R. S. Crim M. J. Riley L. K. Myles M. H. (2016). Influence of rack design and disease prevalence on detection of rodent pathogens in exhaust debris samples from individually ventilated caging systems. J. Am. Assoc. Lab. Anim. Sci. 55, 782–788. 27931317 PMC5113880

[B7] Baxter A. G. Koulmanda M. Mandel T. E. (1991). High and low diabetes incidence nonobese diabetic (NOD) mice: origins and characterisation. Autoimmunity 9, 61–67. doi: 10.3109/08916939108997125 1669848

[B8] Bidot W. A. Ericsson A. C. Franklin C. L. (2018). Effects of water decontamination methods and bedding material on the gut microbiota. PLoS ONE 13:e0198305. doi: 10.1371/journal.pone.0198305 30359379 PMC6201873

[B9] Bolyen E. Rideout J. R. Dillon M. R. Bokulich N. A. Abnet C. C. Al-Ghalith G. A. . (2019). Reproducible, interactive, scalable and extensible microbiome data science using QIIME 2. Nat. Biotechnol. 37, 852–857. doi: 10.1038/s41587-019-0209-9 31341288 PMC7015180

[B10] Brown K. Godovannyi A. Ma C. Zhang Y. Ahmadi-Vand Z. Dai C. . (2016). Prolonged antibiotic treatment induces a diabetogenic intestinal microbiome that accelerates diabetes in NOD mice. ISME J. 10, 321–332. doi: 10.1038/ismej.2015.114 26274050 PMC4737925

[B11] Callahan B. J. McMurdie P. J. Rosen M. J. Han A. W. Johnson A. J. Holmes S. P. (2016). DADA2: high-resolution sample inference from Illumina amplicon data. Nat. Methods 13, 581–583. doi: 10.1038/nmeth.3869 27214047 PMC4927377

[B12] Candon S. Perez-Arroyo A. Marquet C. Valette F. Foray A. P. Pelletier B. . (2015). Antibiotics in early life alter the gut microbiome and increase disease incidence in a spontaneous mouse model of autoimmune insulin-dependent diabetes. PLoS ONE 10:e0125448. doi: 10.1371/journal.pone.0125448 25970503 PMC4430542

[B13] Cani P. D. Depommier C. Derrien M. Everard A. de Vos W. M. (2022). *Akkermansia muciniphila*: paradigm for next-generation beneficial microorganisms. Nat. Rev. Gastroenterol. Hepatol. 19, 625–637. doi: 10.1038/s41575-022-00631-9 35641786

[B14] Caporaso J. G. Lauber Christian L. Walters W. A. Berg-Lyons D. Lozupone C. A. Turnbaugh P. J. . (2011). Global patterns of 16S rRNA diversity at a depth of millions of sequences per sample. *Proc. Natl. Acad. Sci*. USA 108, 4516–4522. doi: 10.1073/pnas.1000080107 PMC3063599 20534432

[B15] Chatzigeorgiou A. Halapas A. Kalafatakis K. Kamper E. (2009). The use of animal models in the study of diabetes mellitus. In Vivo 23, 245–258. 19414410

[B16] Chen B. Chen H. Shu X. Yin Y. Li J. Qin J. . (2018). Presence of segmented filamentous bacteria in human children and its potential role in the modulation of human gut immunity. Front. Microbiol. 9:1403. doi: 10.3389/fmicb.2018.01403 30008704 PMC6034559

[B17] Chen Y. G. Mathews C. E. Driver J. P. (2018). The role of NOD mice in type 1 diabetes research: lessons from the past and recommendations for the future. Front. Endocrinol. 9:51. doi: 10.3389/fendo.2018.00051 29527189 PMC5829040

[B18] Collado M. C. Derrien M. Isolauri E. de Vos W. M. Salminen S. (2007). Intestinal integrity and *Akkermansia muciniphila*, a mucin-degrading member of the intestinal microbiota present in infants, adults, and the elderly. Appl. Environ. Microbiol. 73, 7767–7770. doi: 10.1128/AEM.01477-07 17933936 PMC2168041

[B19] Czech L. Barbera P. Stamatakis A. (2020). Genesis and Gappa: processing, analyzing and visualizing phylogenetic (placement) data. Bioinformatics 36, 3263–3265. doi: 10.1093/bioinformatics/btaa070 32016344 PMC7214027

[B20] de Goffau M. C. Fuentes S. van den Bogert B. Honkanen H. de Vos W. M. Welling G. W. . (2014). Aberrant gut microbiota composition at the onset of type 1 diabetes in young children. Diabetologia 57, 1569–1577. doi: 10.1007/s00125-014-3274-0 24930037

[B21] De Riva A. Wallberg M. Ronchi F. Coulson R. Sage A. Thorne L. . (2017). Regulation of type 1 diabetes development and B-cell activation in nonobese diabetic mice by early life exposure to a diabetogenic environment. PLoS ONE 12:e0181964. doi: 10.1371/journal.pone.0181964 28771521 PMC5542673

[B22] Derrien M. Collado M. C. Ben-Amor K. Salminen S. de Vos W. M. (2008). The Mucin degrader *Akkermansia muciniphila* is an abundant resident of the human intestinal tract. Appl. Environ. Microbiol. 74, 1646–1648. doi: 10.1128/AEM.01226-07 18083887 PMC2258631

[B23] Desai M. Zeggini E. Horton V. A. Owen K. R. Hattersley A. T. Levy J. C. . (2006). The variable number of tandem repeats upstream of the insulin gene is a susceptibility locus for latent autoimmune diabetes in adults. Diabetes 55, 1890–1894. doi: 10.2337/db06-0089 16731859

[B24] Douglas G. M. Maffei V. J. Zaneveld J. R. Yurgel S. N. Brown J. R. Taylor C. M. . (2020). PICRUSt2 for prediction of metagenome functions. Nat. Biotechnol. 38, 685–688. doi: 10.1038/s41587-020-0548-6 32483366 PMC7365738

[B25] Drazenovich N. L. Franklin C. L. Livingston R. S. Besselsen D. G. (2002). Detection of rodent *Helicobacter* spp. by use of fluorogenic nuclease polymerase chain reaction assays. Comp. Med. 52, 347–353. 12211279

[B26] Ericsson A. C. Davis D. J. Franklin C. L. Hagan C. E. (2015). Exoelectrogenic capacity of host microbiota predicts lymphocyte recruitment to the gut. Physiol. Genom. 47, 243–252. doi: 10.1152/physiolgenomics.00010.2015 25852170 PMC4491528

[B27] Ericsson A. C. McAdams Z. L. Dorfmeyer R. A. Hart M. L. O'Neil-Blair A. Amos-Landgraf J. . (2025). Dominant effects of the immediate environment on the gut microbiome of mice used in biomedical research. mSystems 10:e0111225. doi: 10.1128/msystems.01112-25 41222144 PMC12710307

[B28] Fahey J. R. Lyons B. L. Olekszak H. L. Mourino A. J. Ratiu J. J. Racine J. J. . (2017). Antibiotic-associated manipulation of the gut microbiota and phenotypic restoration in NOD mice. Comp. Med. 67, 335–343. 28830580 PMC5557205

[B29] Fallon M. T. Benjamin W. H. Jr. Schoeb T. R. Briles D. E. (1991). Mouse hepatitis virus strain UAB infection enhances resistance to *Salmonella typhimurium* in mice by inducing suppression of bacterial growth. Infect. Immun. 59, 852–856. doi: 10.1128/iai.59.3.852-856.1991 1847697 PMC258337

[B30] Fernandez Trigo N. Kalbermatter C. Yilmaz B. Ganal-Vonarburg S. C. (2024). The protective effect of the intestinal microbiota in type-1 diabetes in NOD mice is limited to a time window in early life. Front. Endocrinol. 15:1425235. doi: 10.3389/fendo.2024.1425235 PMC11464356 39391872

[B31] Filippi C. M. von Herrath M. G. (2008). Viral trigger for type 1 diabetes: pros and cons. Diabetes 57, 2863–2871. doi: 10.2337/db07-1023 18971433 PMC2570378

[B32] Fox J. G. (2002). The non-*H pylori* helicobacters: their expanding role in gastrointestinal and systemic diseases. Gut 50, 273–283. doi: 10.1136/gut.50.2.273 11788573 PMC1773096

[B33] Funda D. P. Kaas A. Bock T. Tlaskalová-Hogenová H. Buschard K. (1999). Gluten-free diet prevents diabetes in NOD mice. Diabetes Metab. Res. Rev. 15, 323–327. doi: 10.1002/(SICI)1520-7560(199909/10)15:5<323::AID-DMRR53>3.0.CO;2-P 10585617

[B34] Godfraind C. Holmes K. V. Coutelier J. P. (1995). Thymus involution induced by mouse hepatitis virus A59 in BALB/c mice. J. Virol. 69, 6541–6547. doi: 10.1128/jvi.69.10.6541-6547.1995 7666556 PMC189556

[B35] Goto Y. Panea C. Nakato G. Cebula A. Lee C. Diez M. G. . (2014). Segmented filamentous bacteria antigens presented by intestinal dendritic cells drive mucosal Th17 cell differentiation. Immunity 40, 594–607. doi: 10.1016/j.immuni.2014.03.005 24684957 PMC4084624

[B36] Grabherr S. Ludewig B. Pikor N. B. (2021). Insights into coronavirus immunity taught by the murine coronavirus. Eur. J. Immunol. 51, 1062–1070. doi: 10.1002/eji.202048984 33687066 PMC8250324

[B37] Gregory G. A. Robinson T. I. G. Linklater S. E. Wang F. Colagiuri S. de Beaufort C. . (2022). Global incidence, prevalence, and mortality of type 1 diabetes in 2021 with projection to 2040: a modelling study. Lancet Diabetes Endocrinol. 10, 741–760. doi: 10.1016/S2213-8587(22)00218-2 36113507

[B38] Hanninen A. Toivonen R. Poysti S. Belzer C. Plovier H. Ouwerkerk J. P. . (2018). *Akkermansia muciniphila* induces gut microbiota remodelling and controls islet autoimmunity in NOD mice. Gut 67, 1445–1453. doi: 10.1136/gutjnl-2017-314508 29269438

[B39] Hart M. L. Ericsson A. C. Franklin C. L. (2017). Differing complex microbiota alter disease severity of the IL-10-/- mouse model of inflammatory bowel disease. Front. Microbiol. 8:792. doi: 10.3389/fmicb.2017.00792 28553262 PMC5425584

[B40] Hart M. L. Ericsson A. C. Lloyd K. C. K. Grimsrud K. N. Rogala A. R. Godfrey V. L. . (2018). Development of outbred CD1 mouse colonies with distinct standardized gut microbiota profiles for use in complex microbiota targeted studies. Sci. Rep. 8:10107. doi: 10.1038/s41598-018-28448-0 29973630 PMC6031694

[B41] Herp S. Durai Raj A. C. Salvado Silva M. Woelfel S. Stecher B. (2021). The human symbiont *Mucispirillum schaedleri*: causality in health and disease. Med. Microbiol. Immunol. 210, 173–179. doi: 10.1007/s00430-021-00702-9 34021796 PMC7615636

[B42] Hillhouse A. E. Myles M. H. Taylor J. F. Bryda E. C. Franklin C. L. (2011). Quantitative trait loci in a bacterially induced model of inflammatory bowel disease. Mamm. Genome 22, 544–555. doi: 10.1007/s00335-011-9343-5 21717222 PMC3804127

[B43] Homberger F. R. (1997). Enterotropic mouse hepatitis virus. Lab. Anim. 31, 97–115. doi: 10.1258/002367797780600189 9175007

[B44] Homberger F. R. Barthold S. W. (1992). Passively acquired challenge immunity to enterotropic coronavirus in mice. Arch. Virol. 126, 35–43. doi: 10.1007/BF01309682 1326267 PMC7086759

[B45] Homberger F. R. Barthold S. W. Smith A. L. (1992). Duration and strain-specificity of immunity to enterotropic mouse hepatitis virus. Lab. Anim. Sci. 42, 347–351. 1331605

[B46] Ivanov I. I. Atarashi K. Manel N. Brodie E. L. Shima T. Karaoz U. . (2009). Induction of intestinal Th17 cells by segmented filamentous bacteria. Cell 139, 485–498. doi: 10.1016/j.cell.2009.09.033 19836068 PMC2796826

[B47] Jain R. Tartar D. M. Gregg R. K. Divekar R. D. Jeremiah Bell J. Lee H.-H. . (2008). Innocuous IFNgamma induced by adjuvant-free antigen restores normoglycemia in NOD mice through inhibition of IL-17 production. J. Exp. Med. 205, 207–218. doi: 10.1084/jem.20071878 18195074 PMC2234380

[B48] Jayaraman S. Jayaraman A. (2018). Long-term provision of acidified drinking water fails to influence autoimmune diabetes and encephalomyelitis. J. Diabetes Res. 2018:3424691. doi: 10.1155/2018/3424691 30035128 PMC6032981

[B49] Jergens A. E. Dorn A. Wilson J. Dingbaum K. Henderson A. Liu Z. . (2006). Induction of differential immune reactivity to members of the flora of gnotobiotic mice following colonization with *Helicobacter bilis* or *Brachyspira hyodysenteriae*. Microbes Infect. 8, 1602–1610. doi: 10.1016/j.micinf.2006.01.019 16698302

[B50] Jergens A. E. Wilson-Welder J. H. Dorn A. Henderson A. Liu Z. Evans R. B. . (2007). *Helicobacter bilis* triggers persistent immune reactivity to antigens derived from the commensal bacteria in gnotobiotic C3H/HeN mice. Gut. 56, 934–40. doi: 10.1136/gut.2006.099242 17145736 PMC1994361

[B51] Jiang H. Q. Bos N. A. Cebra J. J. (2001). Timing, localization, and persistence of colonization by segmented filamentous bacteria in the neonatal mouse gut depend on immune status of mothers and pups. Infect. Immun. 69, 3611–3617. doi: 10.1128/IAI.69.6.3611-3617.2001 11349021 PMC98348

[B52] Kaehler B. D. Bokulich N. A. McDonald D. Knight R. Caporaso J. G. Huttley G. A. (2019). Species abundance information improves sequence taxonomy classification accuracy. Nat. Commun. 10:4643. doi: 10.1038/s41467-019-12669-6 31604942 PMC6789115

[B53] Kassambara A. (2021). rstatix: Pipe-Friendly Framework for Basic Statistical Tests.

[B54] Khanolkar A. Hartwig S. M. Haag B. A. Meyerholz D. K. Epping L. L. Haring J. S. . (2009). Protective and pathologic roles of the immune response to mouse hepatitis virus type 1: implications for severe acute respiratory syndrome. J. Virol. 83, 9258–9272. doi: 10.1128/JVI.00355-09 19570864 PMC2738266

[B55] Körner R. W. Majjouti M. Alcazar M. A. A. Mahabir E. (2020). Of mice and men: the coronavirus MHV and mouse models as a translational approach to understand SARS-CoV-2. Viruses 12:880. doi: 10.3390/v12080880 32806708 PMC7471983

[B56] Kriegel M. A. Sefik E. Hill J. A. Wu H. J. Benoist C. Mathis D. (2011). Naturally transmitted segmented filamentous bacteria segregate with diabetes protection in nonobese diabetic mice. Proc Natl Acad Sci USA. 108, 11548–11553. doi: 10.1073/pnas.1108924108 21709219 PMC3136249

[B57] Kuehl C. J. Wood H. D. Marsh T. L. Schmidt T. M. Young V. B. (2005). Colonization of the cecal mucosa by *Helicobacter hepaticus* impacts the diversity of the indigenous microbiota. Infect. Immun. 73, 6952–6961. doi: 10.1128/IAI.73.10.6852-6961.2005 16177375 PMC1230902

[B58] Kullberg M. C. Andersen J. F. Gorelick P. L. Caspar P. Suerbaum S. Fox J. G. . (2003). Induction of colitis by a CD4+ T cell clone specific for a bacterial epitope. Proc. Natl. Acad. Sci. USA 100, 15830–15835. doi: 10.1073/pnas.2534546100 14673119 PMC307653

[B59] Kyuwa S. Machii K. Okumura A. Toyoda Y. (1996). Characterization of T cells expanded *in vivo* during primary mouse hepatitis virus infection in mice. J. Vet. Med. Sci. 58, 431–437. doi: 10.1292/jvms.58.431 8741603

[B60] Lécuyer E. Rakotobe S. Lengliné-Garnier H. Lebreton C. Picard M. Juste C. . (2014). Segmented filamentous bacterium uses secondary and tertiary lymphoid tissues to induce gut IgA and specific T helper 17 cell responses. Immunity 40, 608–620. doi: 10.1016/j.immuni.2014.03.009 24745335

[B61] Lee S. K. Youn H. Y. Hasegawa A. Nakayama H. Goto N. (1994). Apoptotic changes in the thymus of mice infected with mouse hepatitis virus, MHV-2. J. Vet. Med. Sci. 56, 879–882. doi: 10.1292/jvms.56.879 7865587

[B62] Lenth R. V. (2022). emmeans: Estimated Marginal Means, aka Least-Squares Means.

[B63] Louca S. Doebeli M. (2018). Efficient comparative phylogenetics on large trees. Bioinformatics 34, 1053–1055. doi: 10.1093/bioinformatics/btx701 29091997

[B64] Loy A. Maixner F. Wagner M. Horn M. (2007). probeBase–an online resource for rRNA-targeted oligonucleotide probes: new features 2007. Nucleic Acids Res. 35, D800–D804. doi: 10.1093/nar/gkl856 17099228 PMC1669758

[B65] Lu K. Knutson C. G. Wishnok J. S. Fox J. G. Tannenbaum S. R. (2012). Serum metabolomics in a *Helicobacter hepaticus* mouse model of inflammatory bowel disease reveal important changes in the microbiome, serum peptides, and intermediary metabolism. J. Proteome Res. 11, 4916–4926. doi: 10.1021/pr300429x 22957933 PMC4026094

[B66] Madsen R. Banday V. S. Moritz T. Trygg J. Lejon K. (2012). Altered metabolic signature in pre-diabetic NOD mice. PLoS ONE 7:e35445. doi: 10.1371/journal.pone.0035445 22514744 PMC3326011

[B67] Marietta E. Horwath I. Meyer S. Khaleghi-Rostamkolaei S. Norman E. Luckey D. . (2022). Administration of human derived upper gut commensal prevotella histicola delays the onset of type 1 diabetes in NOD mice. BMC Microbiol. 22:8. doi: 10.1186/s12866-021-02406-9 34983374 PMC8729070

[B68] Mariño E. Richards J. L. McLeod K. H. Stanley D. Yap Y. A. Knight J. . (2017). Gut microbial metabolites limit the frequency of autoimmune T cells and protect against type 1 diabetes. Nat. Immunol. 18, 552–562. doi: 10.1038/ni.3713 28346408

[B69] Martin M. (2011). Cutadapt removes adapter sequences from high-throughput sequencing reads. EMBnet.journal 17. doi: 10.14806/ej.17.1.200

[B70] Mirarab S. Nguyen N. Warnow T. (2011). SEPP: SATé-enabled phylogenetic placement. Pac. Symp. Biocomput. 2012, 247–258. doi: 10.1142/9789814366496_0024 22174280

[B71] Morrison P. J. Bending D. Fouser L. A. Wright J. F. Stockinger B. Cooke A. . (2013). Th17-cell plasticity in *Helicobacter hepaticus*-induced intestinal inflammation. Mucosal Immunol. 6, 1143–1156. doi: 10.1038/mi.2013.11 23462910

[B72] Moskowitz J. E. Andreatta F. Amos-Landgraf J. (2019). The gut microbiota modulates differential adenoma suppression by B6/J and B6/N genetic backgrounds in Apc(Min) mice. Mamm. Genome. 30, 237–244. doi: 10.1007/s00335-019-09814-3 31549210 PMC6842652

[B73] Musaji A. Cormont F. Thirion G. Cambiaso C. L. Coutelier J.-P. (2004). Exacerbation of autoantibody-mediated thrombocytopenic purpura by infection with mouse viruses. Blood 104, 2102–2106. doi: 10.1182/blood-2004-01-0310 15205264

[B74] Musaji A. Meite M. Detalle L. Franquin S. Cormont F. Préat V. . (2005). Enhancement of autoantibody pathogenicity by viral infections in mouse models of anemia and thrombocytopenia. Autoimmun. Rev. 4, 247–252. doi: 10.1016/j.autrev.2004.11.010 15893720 PMC7185387

[B75] Myles M. H. Dieckgraefe B. K. Criley J. M. Franklin C. L. (2007). Characterization of cecal gene expression in a differentially susceptible mouse model of bacterial-induced inflammatory bowel disease. Inflamm. Bowel Dis. 13, 822–836. doi: 10.1002/ibd.20138 17455200

[B76] Myles M. H. Livingston R. S. Livingston B. A. Criley J. M. Franklin C. L. (2003). Analysis of gene expression in ceca of *Helicobacter hepaticus*-infected A/JCr mice before and after development of typhlitis. Infect. Immun. 71, 3885–3893. doi: 10.1128/IAI.71.7.3885-3893.2003 12819073 PMC162032

[B77] Ogle G. D. Wang F. Haynes A. Gregory G. A. King T. W. Deng K. . (2025). Global type 1 diabetes prevalence, incidence, and mortality estimates 2025: results from the International diabetes Federation Atlas, 11th Edition, and the T1D Index Version 3.0. Diabetes Res. Clin. Pract. 225:112277. doi: 10.1016/j.diabres.2025.112277 40412624

[B78] Oksanen J. Simpson G. L. Blanchet F. G. Kindt R. (2022). vegan: Community Ecology Package. Available online at: https://cran.r-project.org/package=vegan (Accessed on October 30, 2024).

[B79] Pakala S. V. Kurrer M. O. Katz J. D. (1997). T helper 2 (Th2) T cells induce acute pancreatitis and diabetes in immune-compromised nonobese diabetic (NOD) mice. J. Exp. Med. 186, 299–306. doi: 10.1084/jem.186.2.299 9221759 PMC2198973

[B80] Paradis E. Schliep K. (2018). ape 5.0: an environment for modern phylogenetics and evolutionary analyses in R. Bioinformatics. 35, 526–528. doi: 10.1093/bioinformatics/bty633 30016406

[B81] Parker S. E. Malone S. Bunte R. M. Smith A. L. (2009). Infectious diseases in wild mice (*Mus musculus*) collected on and around the University of Pennsylvania (Philadelphia) Campus. Comp. Med. 59, 424–430. 19887025 PMC2771607

[B82] Peng J. Narasimhan S. Marchesi J. R. Benson A. Wong F. S. Wen L. (2014). Long term effect of gut microbiota transfer on diabetes development. J Autoimmun. 53, 85–94. doi: 10.1016/j.jaut.2014.03.005 24767831 PMC4361177

[B83] Pociot F. Lernmark A. (2016). Genetic risk factors for type 1 diabetes. Lancet 387, 2331–2339. doi: 10.1016/S0140-6736(16)30582-7 27302272

[B84] Pruesse E. Quast C. Knittel K. Fuchs B. M. Ludwig W. Peplies J. . (2007). SILVA: a comprehensive online resource for quality checked and aligned ribosomal RNA sequence data compatible with ARB. Nucleic Acids Res. 35, 7188–7196. doi: 10.1093/nar/gkm864 17947321 PMC2175337

[B85] R Core Team (2022). R: A Language and Environment for Statistical Computing. Vienna: R Foundation for Statistical Computing.

[B86] Rewers M. Ludvigsson J. (2016). Environmental risk factors for type 1 diabetes. Lancet 387, 2340–2348. doi: 10.1016/S0140-6736(16)30507-4 27302273 PMC5571740

[B87] Rossi M. Hanninen M. L. (2012). Helicobacter spp. other than H. pylori. Helicobacter 17(Suppl 1), 56–61. doi: 10.1111/j.1523-5378.2012.00984.x 22958157

[B88] Rouland M. Beaudoin L. Rouxel O. Bertrand L. Cagninacci L. Saffarian A. . (2022). Gut mucosa alterations and loss of segmented filamentous bacteria in type 1 diabetes are associated with inflammation rather than hyperglycaemia. Gut 71, 296–308. doi: 10.1136/gutjnl-2020-323664 33593807

[B89] Russell A. (2023). “The gut microbiota and targeted pathobiont exposure alters type 1 diabetes phenotype in non-obese diabetic mice (Chapter 4),” in Environmental Variability Affects Gut Microbiota and Experimental Outcomes in Mouse Models. Veterinary Pathobiology (Vol. 147, Columbia, MO: University of Missouri).

[B90] Shoda L. K. Young D. L. Ramanujan S. Whiting C. C. Atkinson M. A. Bluestone J. A. . (2005). A comprehensive review of interventions in the NOD mouse and implications for translation. Immunity 23, 115–126. doi: 10.1016/j.immuni.2005.08.002 16111631

[B91] Singh B. Schwartz J. A. Sandrock C. Bellemore S. M. Nikoopour E. (2013). Modulation of autoimmune diseases by interleukin (IL)-17 producing regulatory T helper (Th17) cells. Indian J. Med. Res. 138, 591–594. 24434314 PMC3928692

[B92] Smith R. (2021). Phytomosaic/ecole: ecole: School of Ecology Package. Aavailable online at: https://github.com/phytomosaic/ecole (Accessed on October 30, 2024).

[B93] Sofi M. H. Gudi R. Karumuthil-Melethil S. Perez N. Johnson B. M. Vasu C. (2014). pH of drinking water influences the composition of gut microbiome and type 1 diabetes incidence. Diabetes 63, 632–644. doi: 10.2337/db13-0981 24194504 PMC3900548

[B94] Stevenson P. G. Doherty P. C. (1998). Kinetic analysis of the specific host response to a murine gammaherpesvirus. J. Virol. 72, 943–949. doi: 10.1128/JVI.72.2.943-949.1998 9444986 PMC124564

[B95] Stough J. M. Dearth S. P. Denny J. E. LeCleir G. R. Schmidt N. W. Campagna S. R. . (2016). Functional characteristics of the gut microbiome in C57BL/6 mice differentially susceptible to *Plasmodium yoelii*. Front. Microbiol. 7:1520. doi: 10.3389/fmicb.2016.01520 27729904 PMC5037233

[B96] Swennes A. G. Sheh A. Parry N. M. Muthupalani S. Lertpiriyapong K. García A. . (2014). *Helicobacter hepaticus* infection promotes hepatitis and preneoplastic foci in farnesoid X receptor (FXR) deficient mice. PLoS ONE 9:e106764. doi: 10.1371/journal.pone.0106764 25184625 PMC4153687

[B97] Talham G. L. Jiang H. Q. Bos N. A. Cebra J. J. (1999). Segmented filamentous bacteria are potent stimuli of a physiologically normal state of the murine gut mucosal immune system. Infect. Immun. 67, 1992–2000. doi: 10.1128/IAI.67.4.1992-2000.1999 10085047 PMC96557

[B98] Trembleau S. Penna G. Bosi E. Mortara A. Gately M. K. Adorini L. (1995). Interleukin 12 administration induces T helper type 1 cells and accelerates autoimmune diabetes in NOD mice. J Exp Med. 181, 817–821. doi: 10.1084/jem.181.2.817 7836934 PMC2191867

[B99] Vatanen T. Franzosa E. A. Schwager R. Tripathi S. Arthur T. D. Vehik K. . (2018). The human gut microbiome in early-onset type 1 diabetes from the TEDDY study. Nature 562, 589–594. doi: 10.1038/s41586-018-0620-2 30356183 PMC6296767

[B100] Velazquez E. M. Nguyen H. Heasley K. T. Saechao C. H. Gil L. M. Rogers A. W. L. . (2019). Endogenous Enterobacteriaceae underlie variation in susceptibility to Salmonella infection. Nat. Microbiol. 4, 1057–1064. doi: 10.1038/s41564-019-0407-8 30911125 PMC6533147

[B101] Villarino N. F. LeCleir G. R. Denny J. E. Dearth S. P. Harding C. L. Sloan S. S. . (2016). Composition of the gut microbiota modulates the severity of malaria. Proc. Natl. Acad. Sci. USA. 113, 2235–2240. doi: 10.1073/pnas.1504887113 26858424 PMC4776451

[B102] Walters W. A. Caporaso J. G. Lauber C. L. Berg-Lyons D. Fierer N. Knight R. (2011). PrimerProspector: *de novo* design and taxonomic analysis of barcoded polymerase chain reaction primers. Bioinformatics 27, 1159–1161. doi: 10.1093/bioinformatics/btr087 21349862 PMC3072552

[B103] Wang Y. Yin Y. Chen X. Zhao Y. Wu Y. Li Y. . (2019). Induction of intestinal Th17 cells by flagellins from segmented filamentous bacteria. Front. Immunol. 10:2750. doi: 10.3389/fimmu.2019.02750 31824516 PMC6883716

[B104] Whary M. T. Morgan T. J. Dangler C. A. Gaudes K. J. Taylor N. S. Fox J. G. (1998). Chronic active hepatitis induced by *Helicobacter hepaticus* in the A/JCr mouse is associated with a Th1 cell-mediated immune response. Infect. Immun. 66, 3142–3148. doi: 10.1128/IAI.66.7.3142-3148.1998 9632578 PMC108325

[B105] Wickham H. (2016). ggplot2: Elegant Graphics for Data Analysis: New York, NY: Springer-Verlag. doi: 10.1007/978-3-319-24277-4_9

[B106] Wilberz S. Partke H. J. Dagnaes-Hansen F. Herberg L. (1991). Persistent MHV (mouse hepatitis virus) infection reduces the incidence of diabetes mellitus in non-obese diabetic mice. Diabetologia 34, 2–5. doi: 10.1007/BF00404016 1647335

[B107] Wolf K. J. Daft J. G. Tanner S. M. Hartmann R. Khafipour E. Lorenz R. G. (2014). Consumption of acidic water alters the gut microbiome and decreases the risk of diabetes in NOD mice. J. Histochem. Cytochem. 62, 237–250. doi: 10.1369/0022155413519650 24453191 PMC3966285

[B108] Wolfe A. E. Moskowitz J. E. Franklin C. L. Wiemken T. L. Ericsson A. C. (2020). Interactions of segmented filamentous bacteria (*Candidatus Savagella*) and bacterial drivers in colitis-associated colorectal cancer development. PLoS ONE 15:e0236595. doi: 10.1371/journal.pone.0236595 32706816 PMC7380633

[B109] Ye Y. Doak T. G. A. (2009). Parsimony approach to biological pathway reconstruction/inference for genomes and metagenomes. PLOS Comput. Biol. 5:e1000465. doi: 10.1371/journal.pcbi.1000465 19680427 PMC2714467

[B110] Yin Y. Wang Y. Zhu L. Liu W. Liao N. Jiang M. . (2013). Comparative analysis of the distribution of segmented filamentous bacteria in humans, mice and chickens. ISME J. 7, 615–621. doi: 10.1038/ismej.2012.128 23151642 PMC3578561

[B111] Yurkovetskiy L. Burrows M. Khan A. A. Graham L. Volchkov P. Becker L. . (2013). Gender bias in autoimmunity is influenced by microbiota. Immunity 39, 400–412. doi: 10.1016/j.immuni.2013.08.013 23973225 PMC3822899

[B112] Zhao Y. Tarbell K. V. (2014). Comment on Sofi et al. pH of Drinking water influences the composition of gut microbiome and type 1 diabetes incidence. Diabetes. 63, 632–644. Diabetes. (2015) 64:e19. doi: 10.2337/db15-0321 24194504 PMC3900548

